# Baseline Amide Proton Transfer Imaging at 3T Fails to Predict Early Response to Induction Chemotherapy in Nasopharyngeal Carcinoma

**DOI:** 10.3389/fonc.2022.822756

**Published:** 2022-02-08

**Authors:** Zhou Liu, Liyan Zou, Qian Yang, Long Qian, Tianran Li, Honghong Luo, Canwen Che, Yuanyuan Lei, Peng Chen, Chunyan Qiu, Xin Liu, Yin Wu, Dehong Luo

**Affiliations:** ^1^ Department of Radiology, National Cancer Center/National Clinical Research Center for Cancer/Cancer Hospital & Shenzhen Hospital, Chinese Academy of Medical Sciences and Peking Union Medical College, Shenzhen, China; ^2^ Paul C. Lauterbur Research Center for Biomedical Imaging, Shenzhen Institute of Advanced Technology, Chinese Academy of Sciences, Shenzhen, China; ^3^ MR Research, GE Healthcare, Beijing, China; ^4^ Department of Pathology, National Cancer Center/National Clinical Research Center for Cancer/Cancer Hospital & Shenzhen Hospital, Chinese Academy of Medical Sciences and Peking Union Medical College, Shenzhen, China; ^5^ Department of Radiation Oncology, National Cancer Center/National Clinical Research Center for Cancer/Cancer Hospital & Shenzhen Hospital, Chinese Academy of Medical Sciences and Peking Union Medical College, Shenzhen, China; ^6^ Department of Radiology, National Cancer Center/National Clinical Research Center for Cancer/Cancer Hospital, Chinese Academy of Medical Sciences and Peking Union Medical College, Beijing, China

**Keywords:** amide proton transfer (APT), nasopharyngeal carcinoma (NPC), Epstein–Barr virus (EBV), clinical stage, induction chemotherapy

## Abstract

**Background:**

Early identification of nasopharyngeal carcinoma (NPC) patients with high risk of failure to induction chemotherapy (IC) would facilitate prompt individualized treatment decisions and thus reduce toxicity and improve overall survival rate. This study aims to investigate the value of amide proton transfer (APT) imaging in predicting short-term response of NPC to IC and its potential correlation with well-established prognosis-related clinical characteristics.

**Methods and Materials:**

A total of 80 pathologically confirmed NPC patients receiving pre-treatment APT imaging at 3T were retrospectively enrolled. Using asymmetry analysis, APT maps were calculated with mean (APT_mean_), 90^th^ percentile (APT_90_) of APT signals in manually segmented NPC measured. APT values were compared among groups with different histopathological subtypes, clinical stages (namely, T, M, N, and overall stages), EBV-related indices (EBV-DNA), or responses to induction chemotherapy, using Mann–Whitney U test or Kruskal–Wallis H test.

**Results:**

NPC showed significantly higher APT_mean_ than normal nasopharyngeal tissues (1.81 ± 0.62% *vs.*1.32 ± 0.56%, *P <*0.001). APT signals showed no significant difference between undifferentiated and differentiated NPC subtypes groups, different EBV-DNA groups, or among T, N, M stages and overall clinical stages of II, III, IVA and IVB (all *P >*0.05). Similarly, baseline APT-related parameters did not differ significantly among different treatment response groups after IC, no matter if evaluated with RECIST criteria or sum volumetric regression ratio (SVRR) (all *P >*0.05).

**Conclusion:**

NPC showed significantly stronger APT effect than normal nasopharyngeal tissue, facilitating NPC lesion detection and early identification. However, stationary baseline APT values exhibited no significant correlation with histologic subtypes, clinical stages and EBV-related indices, and showed limited value to predict short-term treatment response to IC.

## Introduction

The number of the newly diagnosed nasopharyngeal carcinoma (NPC) patients has been reported to be over 129,000 worldwide in 2018, among which 70% is aggregated in East Asia and Southeast Asia (1). In particular, the incidence is as high as 9.69/100,000 in Southeastern China (2). Currently, induction chemotherapy (IC) followed by concurrent chemoradiotherapy (CCRT) has become the standard treatment for locoregionally NPC for its better prognosis than CCRT alone (3, 4). However, around 10% patients still do not respond well to IC. Identification of those non-responders in advance would facilitate more prompt personalized treatment decisions, avoiding chemotherapy toxicity and unnecessary costs. In addition, tumor response to IC has been identified as an independent prognostic factor for long-term survival (5, 6). Hence, pretreatment prediction of short-term response to IC is essential for better individualized management of NPC patients.

Due to substantial biological heterogeneity, NPCs with the same clinical stage might have completely different responses to IC. Identification of biomarkers that could reflect tumor microenvironment characteristics may benefit the prediction of treatment response. With the advancement of morphological and function imaging techniques, MRI has been widely used for NPC evaluation by providing information at both macroscopic and microscopic levels (7–10). Recently, APT-weighted (APT_w_) imaging has emerged as a powerful tool to probe the chemical exchange saturation transfer (CEST) effect between endogenous proteins/peptide amide protons and bulk water (11). Because of overexpressed proteins in tumors, APT_w_ imaging has been increasingly used in tumor detection, grading, and treatment response evaluation of various types of cancer (12–26). Although APT_w_ imaging has shown promising efficacy in predicting middle-term outcome after complete standard treatment (induction chemotherapy followed by concurrent chemoradiotherapy) (26) or 2-year long-term outcome prediction in NPC patients (27), little is known about whether APT signal could predict short-term treatment response to IC only.

Besides, NPC can be classified into distinct subtypes by well-established prognosis-related characteristics, e.g. Epstein–Barr virus (EBV) infection status (28), different histopathological subtypes (29) and clinical stages, etc. It remains unknown whether these distinct NPC subtypes with different prognosis have different protein metabolism levels, which potentially could be quantified by APT_w_ imaging. Therefore, if there is any association between baseline APT measurements and well-established prognosis-related clinical characteristics of NPC, such as histopathological subtypes, clinical stages, and status of Epstein–Barr (EB) virus infection, the value of APT in predicting prognosis would be further strengthened.

In this study, we aimed to investigate the potential value of APT_w_ imaging in predicting short-term response to IC and its potential association with prognosis-related histopathological subtypes, clinical stages, and EBV-related indices.

## Materials and Methods

### Patient Enrollment

This retrospective study was approved by the local ethics committee of the Cancer Hospital & Shenzhen Hospital, Chinese Academy of Medical Sciences, and informed consent was waived. From December 2019 to August 2021, 114 patients with suspected NPC who had undergone baseline APT-weighed imaging were enrolled for further selection. The inclusion and exclusion criteria were as follows: 1) biopsy confirmed NPC; 2) the maximal diameter of the primary nasopharyngeal lesion was larger than 1 cm; 3) conventional MRI and APT-weighted imaging were performed before treatment; 4) each patient had received at least one examination for clinical staging work-up, such as chest X-ray, abdominal ultrasonography, chest and abdomen CT, bone SPECT scan, or whole-body PET-CT scan. Clinical staging for each NPC patient was performed based on the 8th edition of the American Joint Committee on Cancer (AJCC) (30); APT-weighted images were reviewed by a board-certified radiologist (ZL with more than 10 years of experience in head and neck imaging) and those with degraded image quality, namely, overt signal lost, motion artifacts, geometric distortion, were excluded from analysis.

### Treatment Strategy

All NPC patients received standard treatment. For NPC patients with clinical stages of I and II, concurrent chemoradiotherapy or radiotherapy alone was given, whereas IC followed by concurrent chemoradiotherapy were prescribed to patients with clinical stages of III and IV. The brief treatment procedure was: 1) IC (GP regimen): Gemcitabine (1 g/m^2^, Days 1 and 8) + cisplatin (75 mg/m^2^, Day 1); or Paclitaxel (135–175 mg/m^2^, Day 1) + cisplatin (75 mg/m^2^, Day 1), 3 weeks per cycle × 2–3 cycles; 2) Concurrent chemoradiotherapy: cisplatin (30 mg/m^2^, Day 1, once a week for 7 times, iv) and concurrent radiation therapy (in a total of 6.6–7.6 weeks, accumulated dose of 66–78 Gy for primary lesion, while 60–70 Gy for regional lymph nodes). With intensity-modulated radiation therapy being used, conventional 6MV-X fractionated radiotherapy (200 cGy each time × 1 time per day × 5 days per week) was performed, which covers the primary NPC lesion and bilateral cervical lymphatics at risk.

### Follow-Up and Study Endpoint

In this study, the endpoint was set between after IC completion and before next-step concurrent chemoradiotherapy. Standard clinical MRI follow-up was performed at the endpoint for each subject.

### MRI Study

MRI study was performed on a 3T MR scanner (Discovery MR 750w, General Electric) using a 32-channel head and neck joint coil. Local shimming was placed to cover the whole nasopharyngeal carcinoma to reduce static magnetic field inhomogeneity. Referencing to T2-weighted images, a single-slice axial plane covering the largest primary nasopharyngeal carcinoma was selected for APT imaging with chemical shift-selective fat suppression and single-shot fast spin echo (FSE) readout (4 RF saturation pulses with duration of 500 ms for each pulse and without interpulse delay, RF saturation power B_1_ of 2 μT, TR = 6.5 s with minimum TE, slice thickness = 4 mm, field of view = 22 × 22 cm^2^, matrix = 128 × 128). In additional to an unsaturated scan, 27 CEST-weighted images with frequency offsets of 0, ± 0.5, ± 1.0, ± 1.5, ± 2.0, ± 2.5, ± 3.0, ± 3.5, ± 4.5, ± 5.0, ± 5.5, ± 6.0, ± 6.5, and ±7.0 ppm were acquired. The total acquisition time is approximately 4.5 min. A water saturation shift referencing (WASSR) map was collected with B_1_ of 0.5 μT (frequency offsets between ±240 Hz with intervals of 48 Hz) to correct B_0_ inhomogeneity.

### Data Analysis

APT data was analyzed in MATLAB (MathWorks). Z-spectra (S) was normalized by the unsaturated scan (S_0_), interpolated by smoothing splines, and corrected the field inhomogeneity with WASSR (31, 32). Pixel-wise APT-weighted value was calculated using asymmetry analysis of APT_w_ (APT-weighted) = S(−3.5 ppm)/S_0_ − S(3.5 ppm)/S_0_.

Region of interest (ROI) of primary NPC was manually delineated on the unsaturated image by a radiologist (LZ with 6 years of experience in head and neck imaging) using open-source ITK-SNAP software (http://itksnap.org) with referencing conventional anatomical images, namely, T1-weighted, T2-weighted, and contrast enhanced T1-weighted images. Overt hemorrhage, air space, necrosis and vessels were excluded. For patients with tumor confined in one side of nasopharynx without involving the midline of posterior wall of nasopharynx, the normal nasopharyngeal tissue was delineated as well. ROI delineation was then reviewed and approved by a senior radiologist (ZL with more than 10 years of experience in head and neck imaging). APT_w_ signals, namely, the average APT (APT_mean_) and 90^th^ percentile (APT_90_), were obtained from each ROI.

### Treatment Response Evaluation

The largest diameter of NPC was manually measured on the slice with the largest area of lesion on axial T_1_-weighted contrast enhanced images by a board-certified radiologist (LZ with 6 years of experience in head and neck imaging) on the vendor workstation. The measurement was reviewed by a senior radiologist (ZL), with any discrepancies resolved through consensus discussion. Then, the treatment response of primary tumor was determined by calculating the relative change percentage of tumor maximal diameter between pre- and post-treatment, as ΔD = (D_pre-treatment_ − D_post-treatment_)/D_pre-treatment_, and classified as complete response (CR, ΔD = 100%), partial response (PR, ΔD ≥30%), stable disease (SD, −20%< ΔD <30%), and progression disease (PD, ΔD ≤−20%) according to the RECIST criteria version 1.1 (33).

Tumor volume was measured by manually delineating and summing NPC lesion from consecutive 2D slices on contrast-enhanced T_1_-weighted image by LZ and then reviewed by a senior radiologist (ZL). The sum volumetric regression ratio (SVRR) was defined as the tumor volume reduction percentage before and after treatment, and 50% reduction in SVRR was arbitrarily chosen as the cut-off value.

### Histopathology and Epstein–Barr (EB) Virus-Related Indices

The following pathological information were collected: 1) histology of nasopharyngeal carcinoma based on WHO classification, namely, nonkeratinizing squamous cell carcinoma (differentiated subtype and undifferentiated subtype), keratinizing squamous cell carcinoma and basaloid squamous cell carcinoma (34); 2) EBV-encoded small RNA (EBER) status using *in situ* hybridization (ISH). Serum test reports before treatment were also examined to obtain the following information: 1) copy numbers of viral Epstein–Barr deoxyribonucleic acid (EBV-DNA) in plasma samples before treatment, whereas copy numbers of 400 as threshold; 2) serum level of early antigen antibody (EA-IgA); and 3) serum level of viral capsid antibody (VCA-IgA).

### Statistical Analysis

All the statistical analyses were performed using SPSS software v. 26.0 (IBM, Armonk, NY). Paired Student’s t-test was performed to evaluate the difference in APT_w_ signals between normal and cancerous nasopharyngeal tissues. Continuous variables were displayed as median (25th–75th percentile). Mann–Whitney U test was used between the two histopathological subtypes. Kruskal–Wallis H test was conducted to assess any significant differences among groups with different clinical stages or treatment responses. Receiver operating characteristic (ROC) analysis was performed and the area under the curve (AUC) was calculated. The optimal cutoff values were determined by maximizing the sum of sensitivity and specificity using YOUDEN index. Pearson’s correlation analysis was used to evaluate the relationship between APT_w_ measurements and tumor regression rate.

## Results

**Figure 1:**
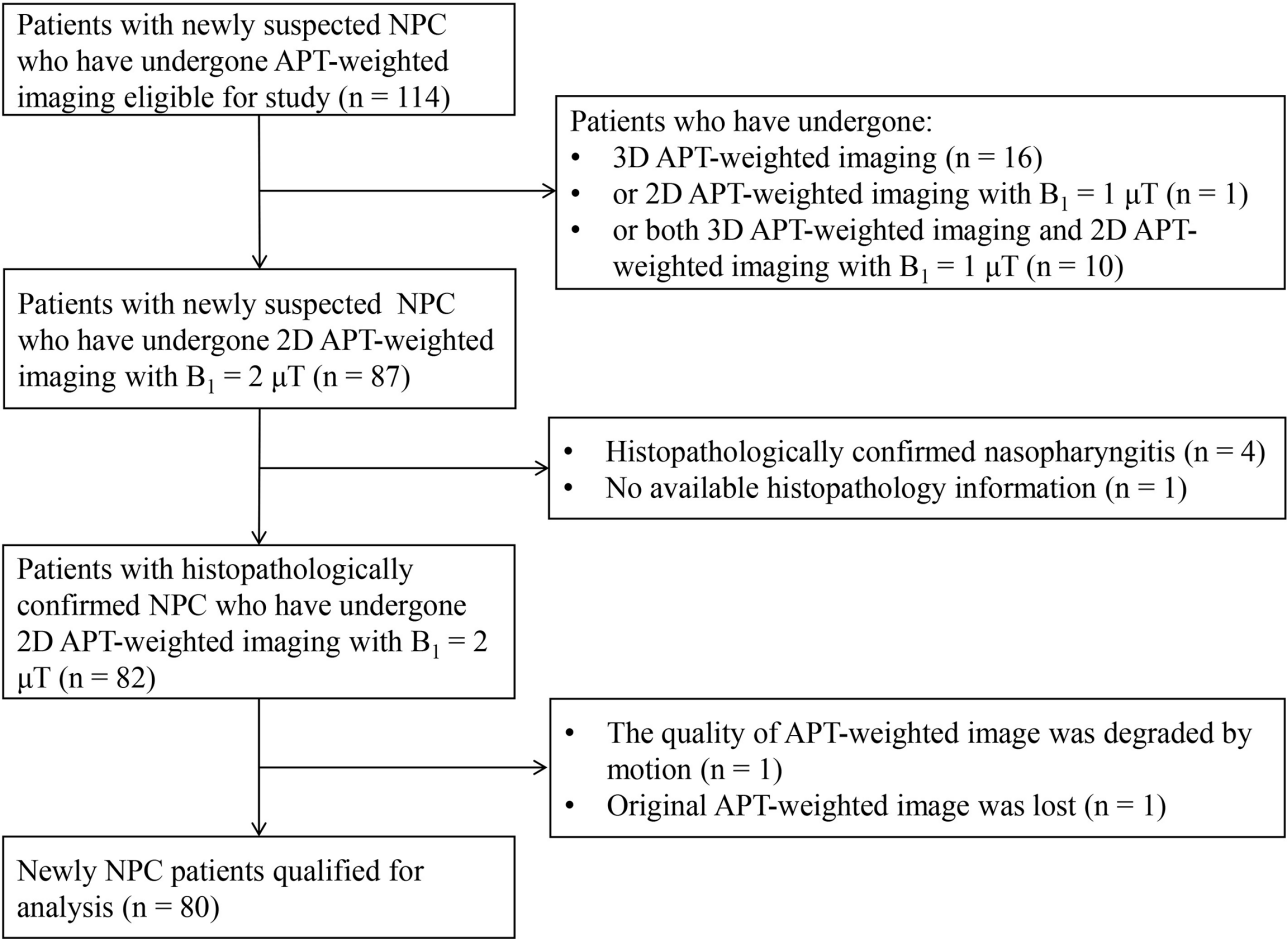
Flowchart of patient enrollment.

A total of 80 patients were included in the analysis (**Figure 1**) with clinical characteristics summarized in **Table 1**.

**Table 1:** Clinical characteristics of the enrolled 80 patients.

Clinical characteristics	Values/No. of patients
Age (years)	41.5 (34-51.25)
Gender	
Male	55
Female	25
Histology	
Nonkeratinizing squamous cell carcinoma	0
Undifferentiated carcinoma	66
Differentiated carcinoma	14
Primary tumor stage	
T1	16
T2	8
T3	41
T4	15
Nodal stage	
N0	1
N1	25
N2	25
N3	29
Metastatic stage	
M0	66
M1	11
Mx	3
Clinical stage	
I	0
II	5
III	33
IVA	27
IVB	12
Not available	3
Treatment strategy	
Radiotherapy only	1
Concurrent chemoradiotherapy	15
Induction chemotherapy and concurrent chemoradiotherapy	58
Treatment strategy not available	6
MRI scan	
Baseline MR scan	80
Follow-up MR scan after induction chemotherapy	53
Follow-up MR scan after radiotherapy only	1
Follow-up MR scan after concurrent chemoradiotherapy	12
No follow-up MR scan	14

### Tissue Characterization

**Figure 2:**
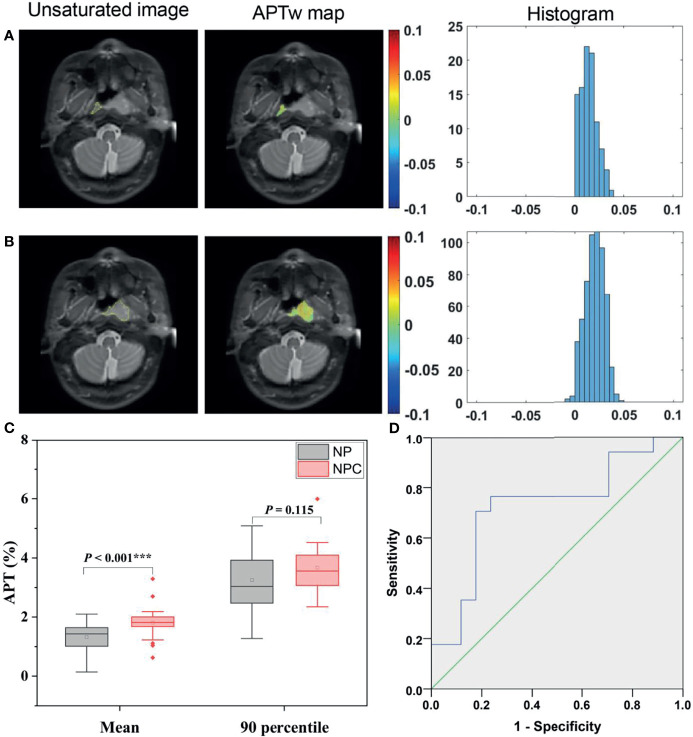
Representative ROI delineation on unsaturated S0 images, APT_w_ maps, and histograms in NPC **(A)** and normal nasopharyngeal tissue **(B)** regions in a 66-year-old male patient. Comparison of APT_w_ signals, including APT_mean_, APT_90_ **(C)** between NPC and corresponding normal nasopharynx tissues; ROC analyses of APT_mean_ in discriminating NPC and normal nasopharynx tissue **(D)**.

A total of 17 patients showed NPC confined in one side and not involving the midline of nasopharynx. **Figures 2A, B** illustrate APT_w_ maps and histograms in NPC and normal nasopharyngeal tissue regions in a 66-year-old male patient. The APT_mean_ of the NPC were significantly higher than that of corresponding normal nasopharyngeal tissue (1.81 ± 0.62% *vs.*1.32 ± 0.56%, *P <*0.001) (**Figure 2C**). However, APT_90_ showed no significant difference between NPC and corresponding normal nasopharyngeal tissue (3.68 ± 0.84% *vs.*3.25 ± 1.07%, *P* = 0.115). The optimal cutoff value of APT_mean_ in discriminating NPC with contralateral nasopharyngeal tissue was 1.66% with a sensitivity of 0.765, a specificity of 0.765, and an AUC of 0.727 (**Figure 2D**).

**Figure 3:**
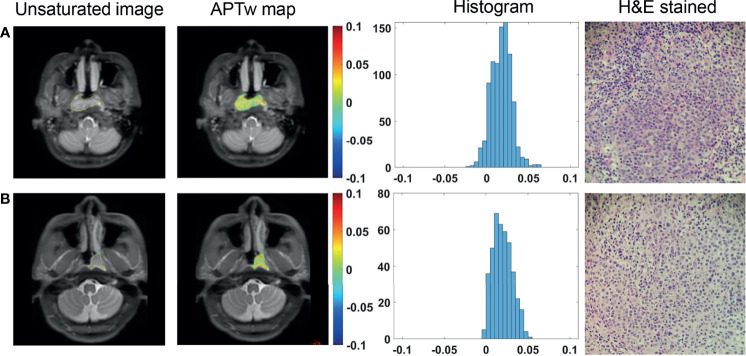
ROI delineation on unsaturated S0 images with ROIs overlaid, APT_w_ maps and histograms, and H&E stained slides (40 × 10) of a 34-year-old female patient with differentiated NPC **(A)** and a 36-year-old male patient with undifferentiated NPC **(B)**.

Among the enrolled 80 patients, 66 had undifferentiated carcinoma, 14 had differentiated carcinoma, and no patients had nonkeratinizing squamous cell carcinoma. All APT_w_ signals did not differ significantly between undifferentiated and differentiated NPC groups (all *P >*0.05) (**Figure 3**), as summarized in **Table 2**.

**Table 2:** APT-related parameters in different histology and TNM stage groups.

	No.	APT_mean_ (%)	APT_90_ (%)
**Nonkeratinizing squamous cell carcinoma**	0	–	–
**Undifferentiated carcinoma**	66	1.72 (1.36–2.10)	3.54 (3.05–4.04)
**Differentiated carcinoma**	14	1.77 (1.45–1.99)	3.20 (3.14–3.77)
***P*-value**	–	0.810	0.695
**Primary tumor stage**			
**T1**	16	1.81 (1.60–2.07)	3.70 (3.46–4.11)
**T2**	8	2.01 (1.72–2.42)	3.67 (3.23–4.52)
** T3**	41	1.64 (1.32–1.96)	3.39 (2.86–3.74)
**T4**	15	1.80 (1.31–2.26)	3.22 (3.03–4.16)
***P*-value**	–	0.330	0.103
**Nodal stage**			
**N0**	1	–	–
**N1**	25	1.76 (1.48–2.00)	3.44 (3.14–4.09)
** N2**	25	1.86 (1.48–2.14)	3.59 (3.06–4.28)
**N3**	29	1.72 (1.32–2.00)	3.45 (3.07–3.82)
***P*-value**	–	0.644	0.867
**Metastatic stage**			
**M0**	66	1.74 (1.48–2.01)	3.42 (3.05–4.03)
**M1**	11	1.53 (1.02–2.21)	3.74 (3.64–4.05)
**Mx^a^**	3	–	–
***P*-value**	–	0.432	0.048*
**Overall tumor stage**			
**I**	0	–	–
**II**	5	1.76 (1.62–1.80)	3.25 (2.89–4.04)
**III**	33	1.86 (1.58–2.14)	3.56 (3.14–4.09)
** IVA**	27	1.59 (1.36–1.91)	3.16 (2.95–3.58)
**IVB**	12	1.62 (1.08–2.18)	3.75 (3.67–4.10)
**Not available**	3	–	–
***P*-value**	–	0.343	0.068

*P < 0.05 indicates significant difference. Mx^a^, metastatic status undetermined.

### TNM Stage

**Figure 4:**
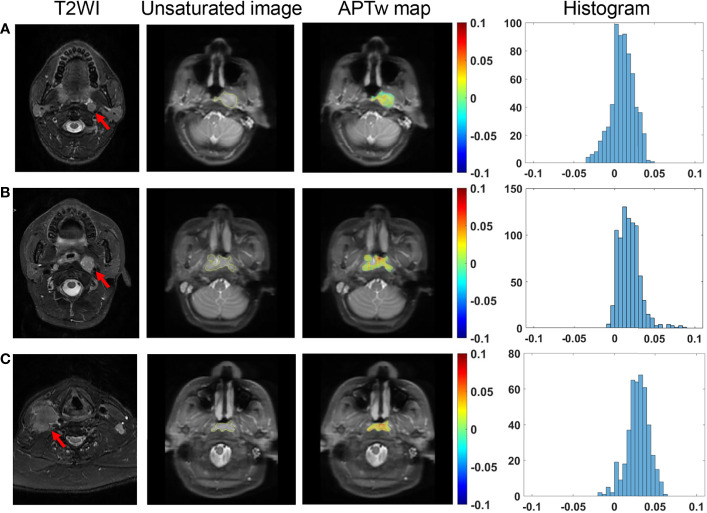
Comparison of T2-weighted images of metastatic lymph nodes (red arrows), and unsaturated S0 images with ROI overlaid, APT_w_ maps and histograms in NPC tissues among different N stages. A 39-year-old male patient with N1 stage NPC **(A)**, a 33-year-old female patient with N2 stage NPC **(B)**, and a 65-year-old male patient with N3 stage NPC **(C)**.

No significant difference was found in APT_w_ signals among primary tumor stages of T1 to T4, among lymph node stages of N1 to N3 (**Figure 4**), between M0 and M1 stages, among overall clinical stages of II, III, IVA, and IVB (all *P >*0.05) (**Table 2**).

### EBV-Related Indices

**Figure 5:**
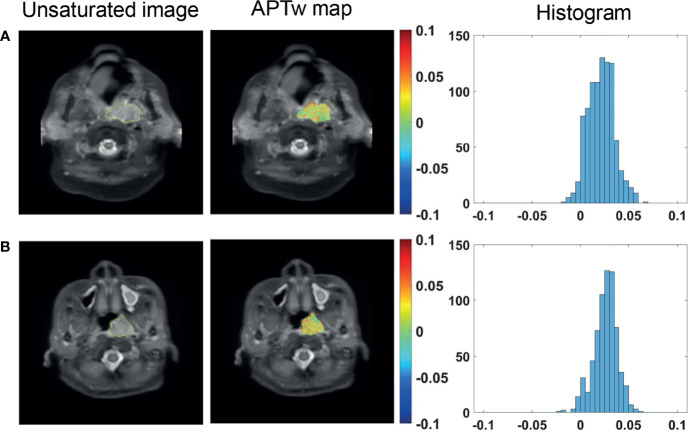
Unsaturated S0 image with ROI overlaid, APT_w_ maps and histograms of a 65-year-old male NPC patient with serum EBV-DNA (+) status **(A)**, and a 47-year-old female NPC patient with serum EBV-DNA (−) status **(B)**.

The 67 patients with available EBER status all showed positive EBER on the specimen using *in situ* hybridization in all cases. For the 76 cases with available serum EBV-DNA quantification, APT_w_ signals was found to be comparable between EBV-DNA positive group and EBV-DNA negative group (**Figure 5**). Similarly, there was no significant difference in APT_w_ signals between EA-IgA positive group (N = 18) and EA-IgA negative group (N = 16), and between VCA-IgA positive group (N = 27) and VCA-IgA negative group (N = 7) (all *P >*0.05) for the 34 NPC patients with available serum expression of EBV antibody (**Table 3**).

**Table 3:** APT-related parameters in groups with different EBV-related indices.

	No.	APT_mean_ (%)	APT_90_ (%)
**EBV-DNA**			
**+**	23	1.72 (1.48–2.04)	3.44 (3.12–4.04)
**−**	53	1.69 (1.28–2.06)	3.45 (3.05–3.78)
**Not available**	4	–	–
***P*-value**	–	0.995	0.730
**EA-IgA**			
**+**	18	1.70 (1.27–1.78)	3.14 (2.94–3.60)
**−**	16	1.84 (1.49–2.20)	3.54 (3.16–4.15)
**Not available**	46	–	–
***P*-value**	–	0.164	0.126
**EB-VCA-IgA**			
**+**	27	1.72 (1.59–1.74)	3.05 (2.92–3.38)
**−**	7	1.80 (1.59–2.00)	3.45 (2.92–4.01)
**Not available**	46	–	–
***P*-value**	–	0.531	0.206
**EBER**			
**+**	67	1.73 (1.40–2.08)	3.45 (3.05–4.03)
**−**	0	–	–
**Not available**	13	–	–

### Treatment Response

**Figure 6:**
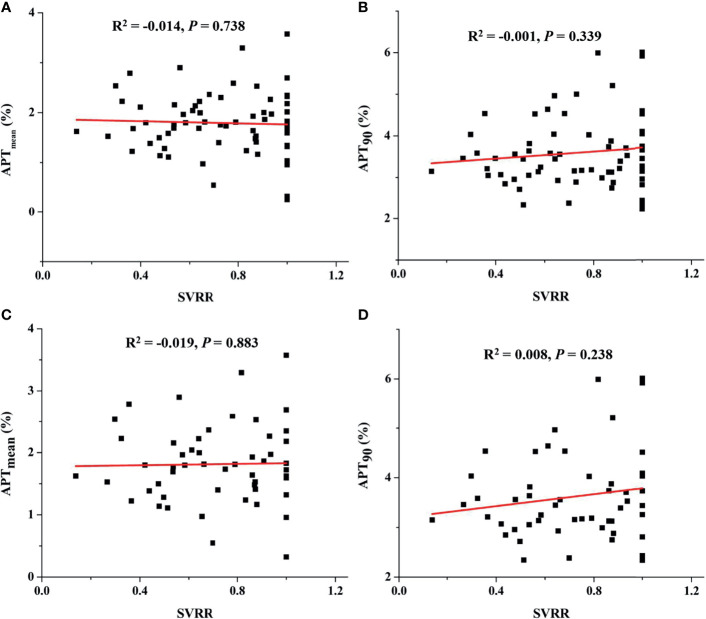
For patients receiving overall treatment, correlation between APT_mean_ and SVRR **(A)**, between APT_90_ and SVRR **(B)**, and for patients who received induction chemotherapy, correlation between APT_mean_ and SVRR **(C)**, between APT_90_ and SVRR **(D)**.

Among 66 patients with available treatment response evaluation after overall treatment, 53 patients had received induction chemotherapy (**Table 4**). For patients receiving overall treatment, no significant correlation was found neither between APT_mean_ and SVRR (R^2^ = −0.014, *P* = 0.738) (**Figure 6A**) nor between APT_90_ and SVRR (R^2^ = −0.001, *P* = 0.339) (**Figure 6B**). Similarly, for patients who received induction chemotherapy, SVRR did not significantly correlate with neither APT_mean_ (R^2^ = −0.019, *P* = 0.883) (**Figure 6C**), nor APT_90_ (R^2^ = 0.008, *P* = 0.238) (**Figure 6D**). Baseline APT-related parameters did not differ significantly among different treatment response groups after overall treatment or after induction chemotherapy, no matter evaluated with the RECIST criteria or SVRR (all *P >*0.05). Besides, no significant difference in tumor regression rate was found between EBV-DNA (+) (n = 16) and EBV-DNA (−) (n = 37) group who received induction chemotherapy, as evaluated with the RECIST criteria (52.0 (38.3–57.2)% *vs.*58.0 (35.0–100.0)%, *P* = 0.407) and SVRR (67.0 (52.5–88)% *vs.* 79.0 (56.0–100.0)%, *P* = 0.441), respectively.

**Table 4:** Baseline APT-related parameters for different treatment response group.

	No.	APT_mean_ (%)	APT_90_ (%)
**Overall treatment response**			
**PD**	0	–	–
**SD**	8	2.11 (1.59–2.36)	3.52 (2.76–4.16)
**PR**	41	1.77 (1.50–2.04)	3.39 (3.13–3.74)
**CR**	17	1.72 (1.32–2.18)	3.65 (2.95–4.10)
***P*-value**	–	0.562	0.880
**Overall treatment response based on SVRR**			
**<50%**	13	1.62 (1.38–2.11)	3.21 (3.05–3.56)
**>50%**	53	1.80 (1.48–2.16)	3.45 (3.13–4.04)
***P*-value**	–	0.514	0.306
**Treatment response after induction chemotherapy**			
**PD**	0	–	–
**SD**	6	2.11 (1.33–2.46)	3.52 (2.65–3.92)
**PR**	35	1.77 (1.45–2.01)	3.25 (3.10–3.78)
**CR**	12	1.72 (1.53–2.22)	3.89 (3.15–4.20)
***P*-value**	–	0.838	0.590
**Treatment response after induction chemotherapy based on SVRR**			
**<50%**	11	1.53 (1.33–2.01)	3.21 (3.01–3.57)
**>50%**	42	1.80 (1.49–2.17)	3.49 (3.13–4.04)
***P*-value**	–	0.380	0.369

## Discussion

In this study, we investigated the value of pre-treatment APT_w_ signal in predicting short-term response after IC in NPC patients and its potential association with prognosis-related clinical characteristics, namely, histopathological subtypes, clinical stages, and EBV-related indices. Unfortunately, based on current result, the stationary baseline APT-weighted imaging showed limited value to predict response to IC and no significant correlation with different histopathological subtypes, EBV infection statuses and clinical stages.

The nasopharynx APT MRI is challenging due to susceptibility artifacts, and swallowing and jaw motion (22, 35). Our image quality was visually comparable to those shown in previous APT studies (22, 35) without apparent signal loss, geometric distortion, or any artifacts. In this study, significantly higher mean APT value in NPC tissue was observed than that in normal nasopharyngeal tissue, which could facilitate NPC lesion detection and early identification. The stronger APT effect may reflect rapidly proliferating tumor cells and abnormally active proteo-synthesis in cancerous tissues, as postulated previously (36).

Although APT-weighted imaging has been proven to be useful in differentiating head and neck malignant and benign tumors (22, 23), it was reported to be difficult in distinguishing malignant tumor types in head and neck regions (23). Our study showed that APT_w_ signals failed to discriminate between undifferentiated and differentiated NPC histology subtypes, which may imply similar protein synthesis status of these two histology subtypes before treatment. As for clinical stages, no significant difference of APT_w_ signals was shown among T, N, and M stages, and overall clinical stages.

EB-virus infection was recognized as the most common etiology of NPC, especially for nasopharyngeal non-keratinizing squamous cell carcinoma in endemic area (37). Consistently, all cases with available EBER *in situ* hybridization were tested EBER positive in our study. The serum EBV-DNA copy number has already been widely used to screen population with the high risk of developing NPC in endemic areas (38) and may serve as a biomarker to monitor and predict treatment response (39). However, pretreatment EBV-DNA status does not show apparent relationship with tumor regression rate after IC in this study. In contrast, a multi-center phase II randomized controlled study showed that an early decrease of EBV-DNA copy number indicates better treatment response (40). This might imply that monitoring the dynamic change of EBV-infection status throughout the treatment course using EBV-DNA copy number might be more effective than merely detecting a stationary pretreatment status in predicting treatment response. However, EBV-DNA copy number quantification data were not available after treatment in this retrospective study. Besides, although it seems no significant correlation of APT signals with EBV-related indices was presented, the correlation between APT_w_ signals change and EBV-DNA copy number alteration should be investigated in the future.

This study focused on the investigation of the value of APT imaging in predicting short-term survival of NPC patients. Although Qamar et al. (26, 27) demonstrated that the mean APT value change between pretreatment and intra-treatment 2 weeks later indeed could predict the middle-term outcome six months after the completion of standard treatment, both pretreatment and intra-treatment mean APT value did not show significant difference between responders and non-responders. Such observations were consistent with our results that no significant difference in pretreatment APT_w_ signals was presented among different treatment response groups. This might also imply that monitoring the dynamic protein change throughout the treatment course using APT_w_ imaging may be more effective than detecting a stationary baseline pretreatment status in predicting treatment response.

Aside from the relatively limited sample size and imbalanced distribution in some groups, several limitations should be noted. First, conventional MTR asymmetry analysis was used to quantify APT effect, which would be contaminated with magnetization transfer and nuclear overhauser enhancement effects. However, the method has been histologically demonstrated to be applicable in tumor characterization (41). In addition, the APT_w_ signal in tumor has proved mostly attributed to the APT contribution under the currently utilized RF power of 2 μT (42). Therefore, the findings regarding APT are valid. Second, this study employed single-slice APT imaging to shorten the scan time, and the limited spatial coverage may be inadequate to characterize intra-tumoral heterogeneity. 3D APT scanning integrated with fast data sampling strategies would be desired for whole-tumor characterization in the future.

### Conclusion

In conclusion, NPC showed significantly stronger APT effect than normal nasopharyngeal tissue, facilitating NPC lesion detection and early identification. However, baseline APT values showed limited value in predicting short-term response to IC and no significant correlation with different histopathological subtypes, EBV infection status, and clinical stages.

## Data Availability Statement

The original contributions presented in the study are included in the article/supplementary material. Further inquiries can be directed to the corresponding authors.

## Ethics Statement

The studies involving human participants were reviewed and approved by the Cancer Hospital & Shenzhen Hospital, Chinese Academy of Medical Sciences. Written informed consent for participation was not required for this study in accordance with the national legislation and the institutional requirements.

## Author Contributions

DL and YW contributed to project idea and supervision and to manuscript revision, and maintained integrity of this manuscript. ZL and LZ implemented the whole study, analyzed the data, and drafted the manuscript. QY and LQ contributed to statistical analysis and reviewed the manuscript. TL, HL, and CC collected the raw MRI data. YL collected the pathological data. PC and CQ collected clinical information. XL provided technical support. All authors listed have made a substantial, direct, and intellectual contribution to the work and approved it for publication.

## Funding

The work described in this paper was supported by grants from the Key Areas Research and Development Program of Guangdong (2019B020235001) and the Shenzhen High-level Hospital Construction Fund.

## Conflict of Interest

Author LQ was employed by GE Healthcare.

The remaining authors declare that the research was conducted in the absence of any commercial or financial relationships that could be construed as a potential conflict of interest.

## Publisher’s Note

All claims expressed in this article are solely those of the authors and do not necessarily represent those of their affiliated organizations, or those of the publisher, the editors and the reviewers. Any product that may be evaluated in this article, or claim that may be made by its manufacturer, is not guaranteed or endorsed by the publisher.

## Abbreviations

NPC, nasopharyngeal carcinoma; APT, amide proton transfer; CEST, chemical exchange saturation transfer; EBV, Epstein–Barr virus; AJCC, American Joint Committee on Cancer; WASSR, water saturation shift referencing; APT_mean_, Mean value of APT asymmetry; APT_90_, 90th percentile of APT asymmetry; RECIST, Response Evaluation Criteria in Solid Tumors; SVRR, sum volumetric regression ratio; EBER, EBV-encoded small RNA; ISH, *in situ* hybridization; EBV-DNA, Epstein–Barr deoxyribonucleic acid; EA-IgA, early antigen antibody immunoglobulin A; VCA-IgA, viral capsid antibody immunoglobulin A; MTR, magnetization transfer ratio.
